# Comparison of vascular and respiratory effects of endothelin-1 in the pig

**DOI:** 10.1155/S0962935193000390

**Published:** 1993

**Authors:** M. G. Clement, M. Dimori, M. Albertini

**Affiliations:** Institute of Veterinary Physiology and Biochemistry University of Milan Via Celoria 10 Milan 20133 Italy

## Abstract

The haemodynamic and respiratory responses caused by i.v. administration of endothelin-1 (ET-1) (20–100 pmol/kg) were studied in anaesthetized spontaneously breathing pigs. Intravenous bolus administration of synthetic ET-1 (40–100 pmol/kg) caused a transient decrease followed by a long-lasting increase in mean pulmonary arterial pressure and dose dependent vasoconstriction both in the systemic and pulmonary circulations. The effect on pulmonary arterial pressure was biphasic, with an initial transient fall followed by a long-lasting dose dependent increase. A biphasic response of the systemic mean arterial pressure was demonstrated only at a high dose of ET-1 (100 pmol/kg). ET-1 administration did not significantly change breathing pattern or phasic vagal input, but caused a significant decrease in passive compliance. Passive resistances or active compliance and resistances of the respiratory system were not modified. These results suggest that in the pig ET-1 is a more potent constrictor of vascular than of bronchial smooth muscle. The vasoconstrictor activity was greater in the pulmonary than the systemic circulations.


Mediators of Inflammation 2, 287–292 (1993)

### Comparison of vascular and respiratory effects of endothelin-1 in the pig

M. G. Clement,[CA] M. Dimori and M. Albertini

Institute of Veterinary Physiology and Biochemistry, University of Milan, Via Celoria 10, 20133 Milan, Italy

[CA] Corresponding Author

**THE haemodynamic and respiratory responses caused by i.v. administration of endothelin-1 (ET-1) (20–100 pmol/kg) were studied in anaesthetized spontaneously breathing pigs. Intravenous bolus administration of synthetic ET-1 (40–100 pmol/kg) caused a transient decrease followed by a long-lasting increase in mean pulmonary arterial pressure and dose dependent vasoconstriction both in the systemic and pulmonary circulations. The effect on pulmonary arterial pressure was biphasic, with an initial transient fall followed by a long-lasting dose dependent increase. A biphasic response of the systemic mean arterial pressure was demonstrated only at a high dose of ET-1 (100 pmol/kg). ET-1 administration did not significantly change breathing pattern or phasic vagal input, but caused a significant decrease in passive compliance. Passive resistances or active compliance and resistances of the respiratory system were not modified. These results suggest that in the pig ET-1 is a more potent constrictor of vascular than of bronchial smooth muscle. The vasoconstrictor activity was greater in the pulmonary than the systemic circulations.**

**Key words:** Bronchoconstrictor activity, Endothelin-1, Pig, Vasoconstrictor activity

### Introduction

Endothelin (ET-1), a 21 amino acid peptide recently isolated from the culture medium of endothelial cells, is one of the most potent vasoconstrictors known.[1] In isolated vascular strips of various experimental animals, ET-1 induces a vasoconstriction that is slow to develop and long-lasting.[2]

ET-1 administered intravenously to healthy volunteers[3] and pigs,[4] had a plasma half-life of about 1 min. Plasma ET-1 was cleared mainly by the kidney, splanchnic circulation and skeletal muscle.[5] Despite its short plasma half-life, the systemic administration of ET-1 to various animal species induces a long-lasting pressure effect *in vivo*, an initial pressure response, either contraction or dilation depending on the species, followed by a secondary vasoconstriction of longer duration.[6–9] The transient hypotensive effect is thought to be mediated by potassium channels[10] and/or by ET-1 evoked release of endothelium derived relaxing factors.[11–15] ET-1 has been shown to contract non-vascular smooth muscle cells also, including guinea-pig tracheal and bronchial strips.[16,17]

*In vivo* potent bronchoconstrictor activity of ET-1 has been demonstrated in the guinea-pig and in the rat.[18,19] Although the haemodynamic activity of ET-1 has been extensively studied in various animal species, very few studies have been performed to evaluate the effects of ET-1 on breathing pattern and compliance and resistances of the respiratory system. Thus, the aim of this investigation was to determine the dose dependence of the haemodynamic and respiratory effects induced by the intravenous administration of ET-1 in the pig. The static and dynamic activities of the tracheobronchial tree were evaluated by studying passive and active pulmonary compliance and resistances.

### Materials and Methods

Six Large White pigs of either sex, weighing 20.5 ± 0.5 kg were used. Animals were sedated with 0.05 ml/kg 1% propionylpromazine hydrochloride, i.m. (Bayer Italia, Spa Milano) and anaesthetized with thiopental sodium (15 mg/kg), i.v., first injection, followed by continuous infusion (9 mg/kg/h) (Farmitalia, Carlo Erba Milano). The spontaneously breathing animals were tied down supine and body temperature was measured with a rectal probe and maintained at 37–38°C by an electric blanket. A tracheal cannula was inserted into the lower portion of the extrathoracic trachea and connected to a Fleish pneumotachograph no. 2 to record the respiratory airflow ($\dot{V}$) and, by electronic integration, tidal volume ($V_T$). A side port of the tracheal cannula was connected to a pressure transducer (Statham 15299) for measurement of tracheal pressure ($P_{tr}$).

© 1993 Rapid Communications of Oxford Ltd

The right femoral artery was cannulated with a polyethylene catheter to monitor arterial blood pressure. The right external jugular vein was cannulated to administer ET-1. A balloon-tipped catheter (Swan–Ganz 5F) was introduced into the pulmonary artery to monitor pulmonary arterial pressure and cardiac output (CO) was evaluated by the thermodilution technique (Cardiac Output Computer 701 I.L.). Systemic and pulmonary arterial pressure were recorded by connecting the catheters to a fluid filled capacitance manometer (Bell & Howell 4-422). All signals were recorded simultaneously on a multichannel pen recorder (Nec San-ei Instruments Polygraph mod. 8K40).

After recording the spontaneous resting breathing pattern the passive compliance was evaluated by occluding the tracheal cannula at the end of inspiration and at various lung volumes during expiration, in order to plot the passive pressure–volume (P–V) relationships of the respiratory system. At each volume, changes in pressure were computed after 0.5 s, a time that seemed to be sufficient to accommodate most of the stress relaxation phenomena. The resistances of the respiratory system (Rrs) were obtained from the $\tau$rs/Crs ratio. The passive time constant of the respiratory system was computed from the expiratory trace flow obtained after reopening the airways closed at the end of inspiration. $\tau$rs was the time interval from the peak expiratory flow to 64% of its decay. The resistance of the respiratory system was calculated by subtracting the resistance value of the set-up. Active compliance (C′rs) and active resistances (R′rs) of the respiratory system were evaluated by occluding the airway at the end-expiratory lung volume to obtain tracheal occlusion pressure. $P_{tr}$, $\dot{V}$ and $V_T$ were measured at 0.04 s intervals after the onset of inspiration, the first during occluded inspiratory effort and the $\dot{V}$ and $V_T$ during the immediately preceding spontaneous inspiration. Onset of inspiration was defined as inspiratory flow and/or a negative deflection in $P_{tr}$. Timing of breathing was analysed in terms of duration of inspiration and expiration for spontaneous ($T_I$, $T_E$) and occluded breaths obtained by occlusion of the airway at the end-expiratory level, as suggested by Miserocchi *et al.*[20,21] The latter manoeuvre provides evaluation of the timing of breathing in the absence of lung volume related vagal afferents. The vagal inhibitory effect on respiratory centres was evaluated as the $T_{I°}/T_I$ ratio. Heart rate (HR), mean pulmonary arterial pressure (MPAP), mean systemic arterial pressure (MAP), total pulmonary vascular resistances (TPVR), and total systemic vascular resistances (TSVR) were also evaluated.

Endothelin-1 (ET-1) (Sigma Chemical Company, St Louis, MO, USA) was dissolved in saline solution and stored at −20°C in a stock solution of 50 μg/ml. Each animal was given 20–100 pmol/kg ET-1 injected as a bolus into the jugular vein. The bronchoconstrictor effect of ET-1 was also evaluated at the concentration of 400 pmol/kg ET-1. In all pigs, the cardiovascular and respiratory parameters were monitored before and continuously after ET-1 injection for 5 min and again every 5 min until 30 min.

The results are expressed as means ± S.E.M. The statistical significance was evaluated by paired Student's *t*-test comparing the value obtained after ET-1 administration to the last pre-injection values. Difference of the values were considered statistically significant at $p < 0.05$.

### Results

As shown in Fig. 1A, the intravenous bolus administration of ET-1 at 40 and 100 pmol/kg to pigs caused long-lasting increases in mean pulmonary arterial pressure (MPAP) that were statistically significant at 15 min and persisted up to 30 min. In contrast, administration of 20 pmol/kg ET-1 did not significantly affect MPAP. At all doses, the pulmonary vasoconstriction was preceded by a transient decrease in MPAP that was completely reversed at 5 min. At 40 pmol/kg, there was a statistically significant increase in total pulmonary vascular resistance (TPVR) (Fig. 1B).

As shown in Fig. 1C, there was a statistically significant increase in mean systemic arterial pressure (MAP) only after the administration of 100 pmol/kg ET-1. The peptide did not significantly affect total systemic vascular resistances (TSVR). The administration of ET-1 did not cause any significant changes in cardiac output (CO) or stroke volume (SV) (Table 1). At 100 pmol/kg, the peptide caused an increase in heart rate that was statistically significant at 100 min. ET-1 did not significantly change respiratory frequency, tidal volume, pulmonary ventilation, inspiratory or expiratory duration of unoccluded breaths (data not shown) at any of the doses.

As shown in Fig. 2A, 40 pmol/kg ET-1 caused a significant lengthening of expiratory time of occluded breaths ($T_{E°}$). In contrast, the lengthening of $T_{I°}$ was not statistically significant (Fig. 2B). Both 20 and 100 pmol/kg of the peptide had no statistically significant effect on these parameters. In control conditions the $T_{I°}/T_I$ ratio, considered to be an index of phasic vagal feed-back, was greater than 1 and it was not significantly changed by ET-1 administration (Fig. 2C).

As shown in the Fig. 3A, the lower doses (20–40 pmol/kg) of ET-1 caused significant and progressive decreases in Crs that were not correlated with increases in passive respiratory

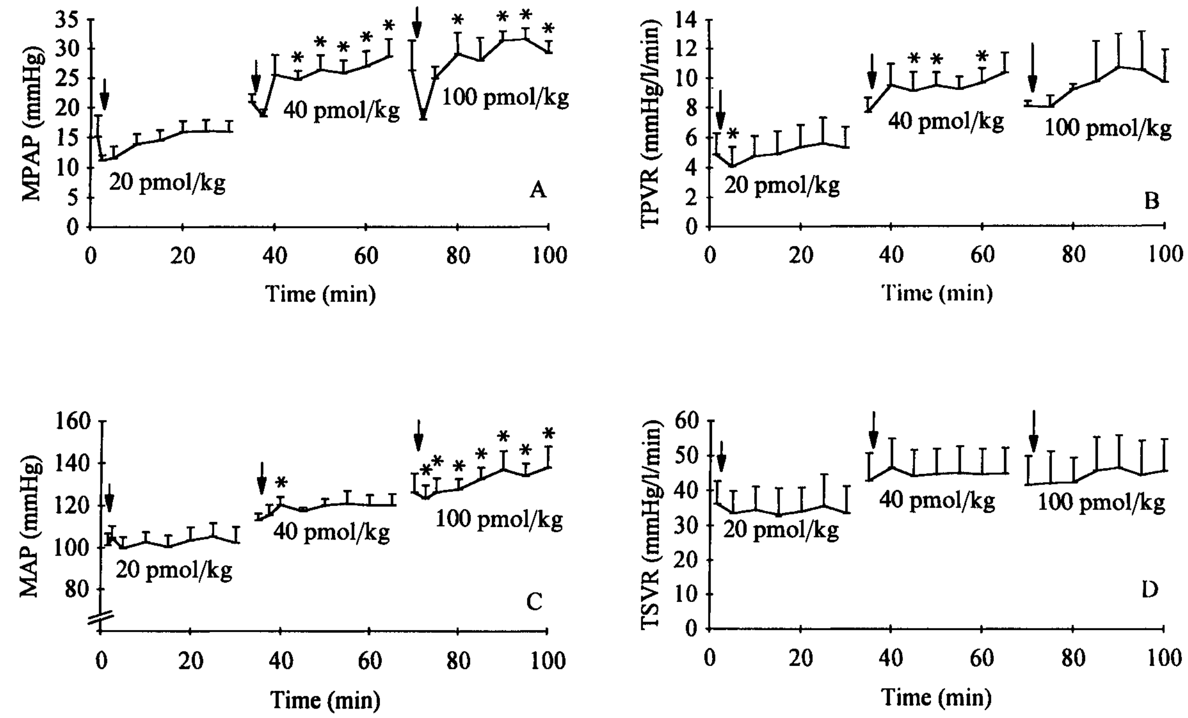

FIG. 1. Pulmonary and systemic vascular responses to bolus ET-1 administration, (20 pmol/kg, 40 pmol/kg, 100 pmol/kg). MPAP, mean pulmonary arterial pressure; TPVR, total pulmonary vascular resistance; MAP, mean systemic arterial pressure; TSVR, total systemic vascular resistance. Results are compared with last pre-injection value and expressed as means ±S.E.M., $n = 6$. The asterisks indicate statistically significant differences (*$p < 0.05$). The arrows indicate bolus ET-1 administration.

resistances (Fig. 3B), while 100 pmol/kg of ET-1 did not change the passive compliance or resistances of the respiratory system (Figs 3A and B). The active compliance (C′rs) and resistances were not significantly affected by ET-1 administration (Figs 3C and D).

In order to demonstrate the bronchoconstrictor activity of the peptide, ET-1 was administered to one pig at a higher concentration (400 pmol/kg). This dose seemed to increase active respiratory resistances without affecting C′rs (data not shown). On the contrary, 400 pmol/kg of ET-1 did not have any greater effects on MPAP and TPVR than those of 40 and 100 pmol/kg (Figs 4A and B).

**Table 1.** Time course of cardiovascular variables in response to i.v. administration of ET-1

| Concentration of ET-1 (pmol/kg) | Time (min) | HR | | CO | | SV | |
|---|---|---|---|---|---|---|---|
| | | Mean | S.E.M. | Mean | S.E.M. | Mean | S.E.M. |
| 20 | 0 (control) | 105 | 8 | 3.0 | 0.4 | 28 | 2 |
| | 5 | 110 | 11 | 3.2 | 0.4 | 29 | 1 |
| | 30 | 120 | 24 | 3.4 | 0.6 | 29 | 2 |
| 40 | 35 (control) | 111 | 15 | 3 | 0.5 | 26 | 1 |
| | 40 | 108 | 11 | 2.8 | 0.4 | 26 | 1 |
| | 65 | 112 | 13 | 2.9 | 0.4 | 26 | 1 |
| 100 | 70 (control) | 130 | 14 | 3.2 | 0.5 | 25 | 3 |
| | 75 | 130 | 16 | 3.2 | 0.6 | 25 | 3 |
| | 100 | 143* | 9 | 3.2 | 0.5 | 23 | 3 |

Results are means ±S.E.M., $n = 6$. Heart rate (HR), cardiac output (CO) and stroke volume (SV). Results are compared with last pre-injection value. *$p < 0.05$.

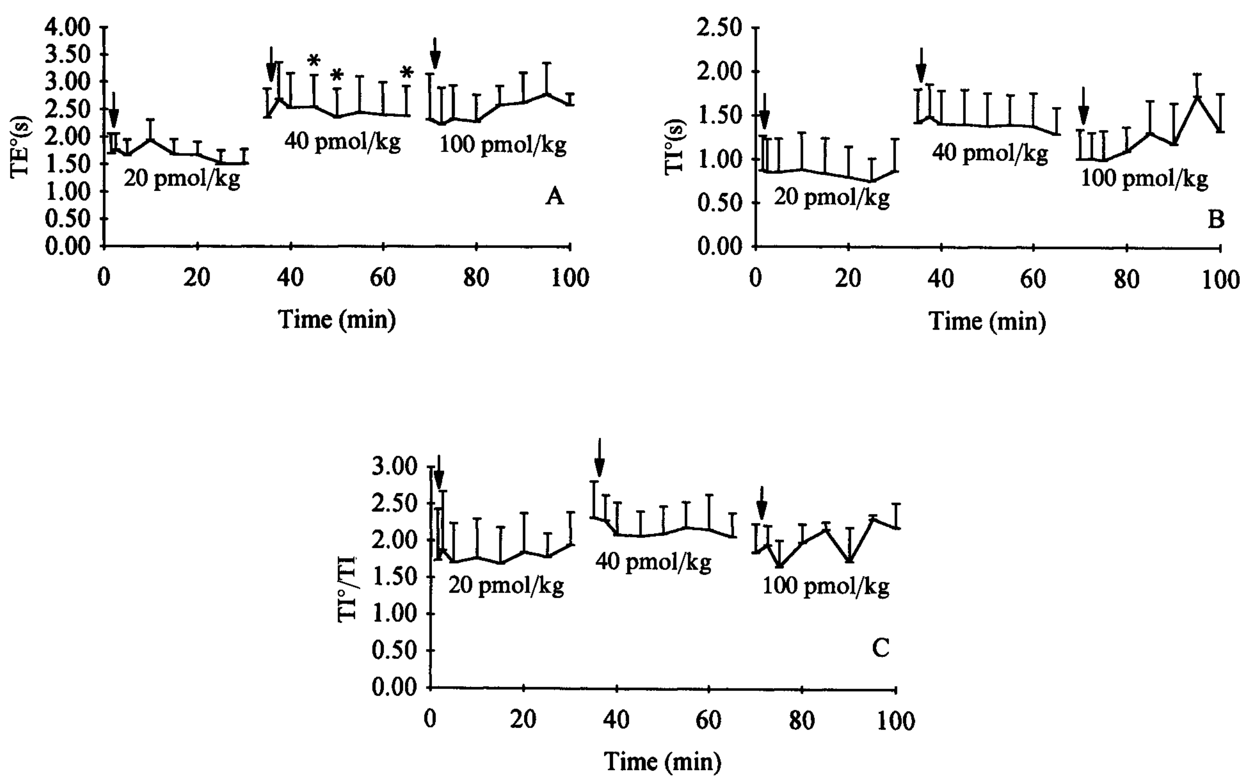

FIG. 2. Respiratory responses to bolus ET-1 administration (20 pmol/kg, 40 pmol/kg, 100 pmol/kg) $T_{E^\circ}$ and $T_{I^\circ}$ represent expiratory (panel A) and inspiratory time (panel B) of occluded breaths. In panel C, $T_{I^\circ}/T_I$ is the ratio of inspiratory times of occluded and unoccluded breaths. Results are compared with last pre-injection value and expressed as means ±S.E.M., $n = 6$. The asterisks indicate statistically significant differences ($^*p < 0.05$). The arrows are as in Fig. 1.

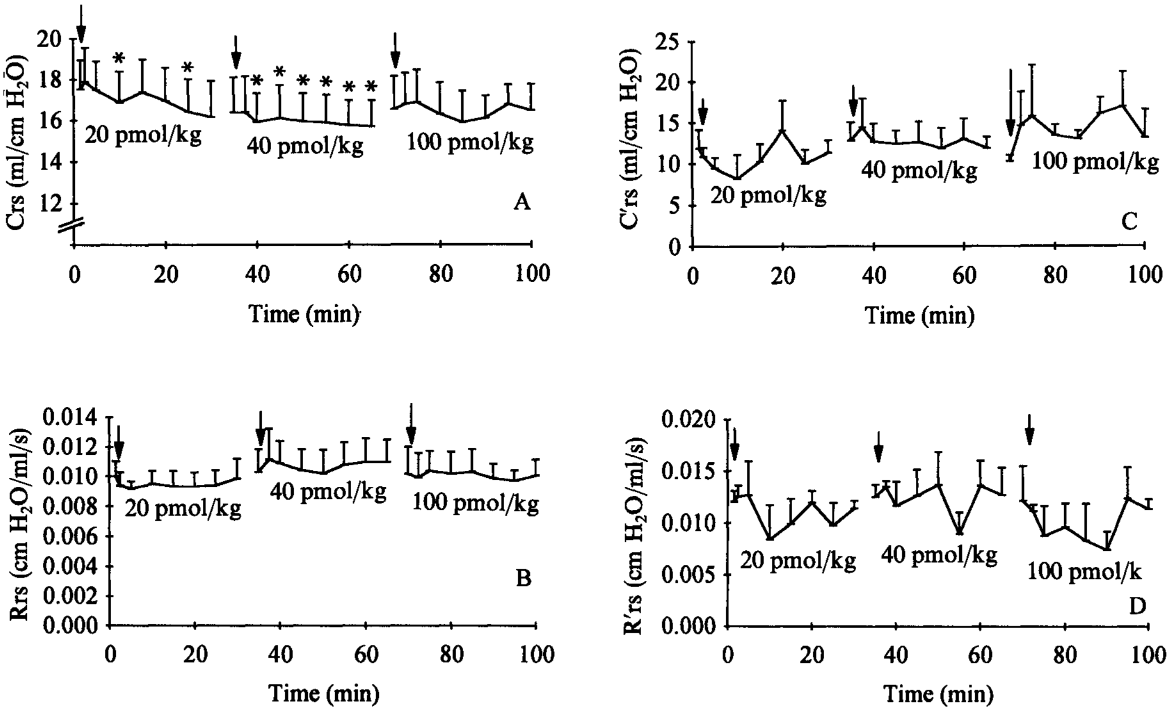

FIG. 3. Time-course of passive compliance (Crs) (panel A) and active compliance (C'rs) (panel C) of the respiratory system, and passive (Rrs) and active resistance (R'rs), (panels B and D, respectively) in response to bolus ET-1 administration. Results are compared with last pre-injection value and expressed as means ±S.E.M., $n = 6$. The asterisks indicate statistically significant differences ($^*p < 0.05$). The arrows are as in Fig. 1.

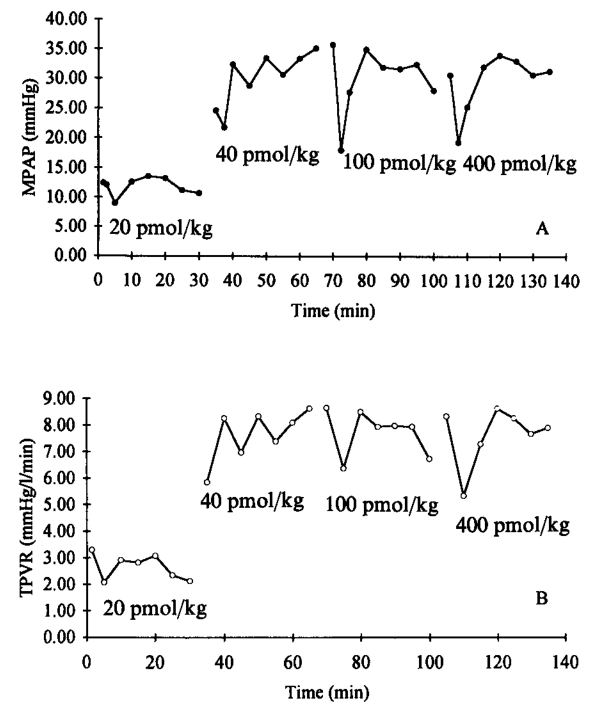

FIG. 4. Time-course of pulmonary arterial pressure (MPAP) (panel A) and total pulmonary vascular resistance (TPVR) (panel B) for one pig, after ET-1 administration.

### Discussion

This study examined the haemodynamic and respiratory effects of ET-1 in anaesthetized and spontaneously breathing pigs. The results show that the peptide is a potent vasoconstrictor mainly for the pulmonary circulation, and a mild bronchoconstrictor. ET-1 bolus administration increased pulmonary arterial pressure and vascular resistances. A maximum effect was obtained at 40 pmol/kg of ET-1. The failure of a higher dose to induce more marked effects is probably due to saturation of lung ET-1 receptors by repeated administration of the peptide to the same animals. After all doses there was an initial transient vasodilation followed by a long-lasting vasoconstriction. In the systemic vascular bed, ET-1 caused a similar biphasic response only at 100 pmol/kg. The biphasic vascular response has been previously demonstrated in various animal species and in the pig by Pernow *et al.*[4] The long-lasting vasoconstrictive activity may be due to irreversible interaction of ET-1 with its specific receptors.[1] The greater vascularization and high density of ET-1 receptors in the lung might be responsible, at least in part, for the greater response of the pulmonary than of the systemic circulation.

At 100 pmol/kg significant vasoconstriction was found in the systemic circulation. Saturation of ET-1 binding sites in the lung by previous administration of the peptide might reduce its pulmonary clearance thus favouring the systemic effect.[4] The transient hypotensive effect in both the pulmonary and systemic circulations may reflect ET-1 induced release of such vasodilators as NO and $PGI_2$.[11–15]

At variance with other animal species, ET-1 did not alter cardiac activity in the pig to any statistically significant extent. The late increase in heart rate, not correlated with the pressure effect, is probably due to the release of ET-1 dependent vasoactive mediators.[11–15]

The results show that ET-1 does not change the breathing pattern or the $T_{I^\circ}/T_I$ ratio, in the pig, but causes a significant lengthening of $T_{E^\circ}$. Because the $T_{I^\circ}/T_I$ ratio is an index of vagal inhibitory activity on respiratory centres, the results show that ET-1 does not alter the vagal input, but causes a change in the bulbo-pontine rhythm probably by reduction of cerebral vascular flow.[22,23] The decrease in Crs was not correlated with a change in passive resistances observed in pigs, which suggests that ET-1 (20–100 pmol/kg) does not alter bronchomotor tone but causes only a decrease in lung distensibility, probably through increases in MPAP, or in lung capillary permeability. The results show that ET-1 does not statistically significantly change C′rs and R′rs probably because of induction of various compensatory mechanisms of both nervous and vascular origin.[24]

Studies have shown that ET-1 acts as a potent bronchoconstrictor when studied *in vitro*[16,17] or *in vivo* in guinea-pigs.[18] In contrast, our results demonstrate that in spontaneously breathing pigs ET-1 is a mild bronchoconstrictor. In fact, constrictive activity on bronchial smooth muscle was caused only by a very high dose of ET-1 (400 pmol/kg). The reason for these discrepancies is not apparent. However, differences in ET-1 catabolism by the lungs of different animal species might be involved.

In summary, the results suggest that the effects on the vascular and respiratory system are different after ET-1 is administered i.v. to spontaneously breathing pigs. ET-1 is a more potent constrictor of vascular than of bronchial smooth muscle. Its vasoconstrictive effect is more marked in the pulmonary than in the systemic vascular bed. Pharmacological studies with selective inhibitors of ET-1 biosynthesis or action should help to clarify the role of ET-1 in the development of pulmonary disease.

### References

1. Yanagisawa M, Kurihara H, Kimura S *et al.* A novel potent vasoconstrictor peptide produced by vascular endothelial cell. *Nature (Lond)* 1988; **332**: 411–415.
2. Clarke JG, *et al.* Endothelin-1 is a potent long-lasting vasoconstrictor in dog peripheral vasculature *in vivo*. *J Cardiovasc Pharmac* 1989; **13**(Suppl.5): S211–S212.

3. Vierhapper H, Waner O, Nowotny P, Waldhausel W. Effect of endothelin-1 in man. *Circulation* 1990; **81**; 1415–1418.
4. Pernow J, Franco-Cereceda A, Matran R, Lundberg JM. Effect of endothelin-1 on regional vascular resistances in the pig. *J Cardiovasc Pharmac* 1989; **13**(Suppl.5): S205–S206.
5. Pernow J, Hemsen A, Lundberg JM. Tissue specific distribution, clearance and vascular effects of endothelin in the pig. *Biochem Biophys Res Commun* 1989; **161**: 647–653.
6. Raffestin B, Adnot S, Eddahibi S, Macquin-Mavier I, Braquet P, Chabrier P. Pulmonary vascular response to endothelin in rats. *J Appl Physiol* 1991; **70**: 567–574.
7. Lippton HL, Hauth TA, Summer WR, Hyman AL. Endothelin produces pulmonary vasoconstriction and systemic vasodilation. *J Appl Physiol* 1989; **66**: 1008–1012.
8. Lippton HL, Ohlstein EH, Summer WR, Hyman AL. Analysis of response to endothelins in the rabbit pulmonary and systemic vascular bed. *J Appl Physiol* 1991; **70**: 331–341.
9. Hoffman A, Grossman E, Ohman KP, Marks E, Keisr HR. The initial vasodilation and the later vasoconstriction of endothelin-1 are selective to specific vascular beds. *Am J Hyperten* 1990; **3**: 789–791.
10. Lippton HL, Cohen GA, McMurtry IF, Hyman AL. Pulmonary vasodilation to endothelin isopeptides *in vivo* is mediated by potassium channel activation. *J Appl Physiol* 1991; **70**: 947–952.
11. Boulanger C, Luscher TF. Release of endothelin from the porcine aorta: inhibition by endothelium-derived nitric oxide. *J Clin Invest* 1990; **85**: 587–590.
12. Luscher TF, Yang Z, Tschudi M, *et al.* Interaction between endothelin-1 and endothelium-derived relaxing factor in human arteries and veins. *Circ Res* 1990; **66**: 1088–1094.
13. Rakugi H, Nakamaru M, Tabuchi Y, Nagano M, Mikami H, Ogihara T. Endothelin stimulates the release of prostacyclin from rat mesenteric arteries. *Biochem Biophys Res Commun* 1989; **160**: 924–928.
14. Thiemermann C, Lidbury PS, Thomas GR, Vane JR. Endothelin-1 releases prostacyclin and inhibits *ex vivo* platelet aggregation in the anaesthetized rabbit. *J Cardiovasc Pharmacol* 1989; 13(Suppl. 5): S138–S141.
15. Rubanyi GM. Endothelium-derived vasoconstrictor factors: an overview. In: Ryan US, Rubanyi GM, eds. *Endothelial regulation of vascular tone.* New York: Marcel Dekker Inc. 1992; 375–386.
16. Maggi CA, Patacchini R, Giuliani S, Meli A. Potent contractile effect of endothelin in isolated guinea-pig airways. *Eur J Pharmacol* 1989; **160**: 179–182.
17. White SR, Hathaway DP, Umans JG, Leff AR. Direct effects on airway smooth muscle contractile response caused by endothelin-1 in guinea-pig trachealis. *Am Rev Respir Dis* 1992; **145**: 491–493.
18. White SR, Hathaway DP, Umans JG, Tallet J, Abrahams C, Leff AR. Epithelial modulation of airway smooth muscle response to endothelin-1. *Am Rev Respir Dis* 1991; **144**: 373–378.
19. Matsuse T, Fukuchi Y, Suruda T, Nagase T, Ouchi Y, Orimo H. Effect of endothelin-1 on pulmonary resistance in rats. *J Appl Physiol* 1990; **68(6)**: 2391–2393.
20. Miserocchi M, Quinn B. Control of breathing during acute haemorrhage in anaesthetized cats. *Respir Physiol* 1980; **41**: 289–305.
21. Miserocchi G, Trippenbach M, Mazzarelli M, Jaspar N, Hazucha M. The mechanism of rapid shallow breathing due to histamine and phenyldiguanide in cats and rabbit. *Respir Physiol* 1978; **32**: 141–153.
22. Shigeno T, Mima T, Takakura K, Yanagisawa M, Saita A, Goto K, Masaki T. Endothelin-1 acts in cerebral arteries from the adventitial but not from the luminal side. *J Cardiovasc Pharmac* 1989; **13**(Suppl.5): S174–S176.
23. Harmstead WM, Mirro R, Leffler CW, Busija DW. Influence of endothelin on piglet cerebral microcirculation. *Am J Physiol* 1989; **257(H26)**: H707–H710.
24. Lloyd TC jr. Cardiopulmonary baroreflexes: effects of pulmonary congestion and edema. *J Appl Physiol* 1977; **43(1)**: 107–113.

**Received 11 March 1993; accepted in revised form 10 May 1993**
